# Innovative epitopes in *Staphylococcal* Protein-A an immuno-informatics approach to combat MDR-MRSA infections

**DOI:** 10.3389/fcimb.2024.1503944

**Published:** 2025-01-14

**Authors:** Pengjun Zhou, Xing Shi, Jinquan Xia, Yifei Wang, Shaowei Dong

**Affiliations:** ^1^ Department of Pharmacology, Guangdong Pharmaceutical University, Guangzhou, China; ^2^ Guangzhou Jinan Biomedicine Research and Development Center, Guangdong Provincial Key Laboratory of Bioengineering Medicine, College of Life Science and Technology, Jinan University, Guangzhou, China; ^3^ Department of Haematology and Oncology, Shenzhen Children’s Hospital, Shenzhen, China

**Keywords:** human leukocyte antigen, immuno-informatics, methicillin-resistant *Staphylococcus aureus*, *Staphylococcal* Protein A, toll-like receptor, vaccine development

## Abstract

**Background:**

Methicillin-resistant *Staphylococcus aureus* (MRSA) poses a significant challenge in clinical environments due to its resistance to standard antibiotics. *Staphylococcal* Protein A (SpA), a crucial virulence factor of MRSA, undermines host immune responses, making it an attractive target for vaccine development. This study aimed to identify potential epitopes within SpA that could elicit robust immune responses, ultimately contributing to the combat against multidrug-resistant (MDR) MRSA.

**Methods:**

The SpA protein sequence was retrieved from the UniProt database, with various bioinformatics tools employed for epitope prediction. T-cell epitopes were identified using the Tepitool server, focusing on high-affinity interactions with prevalent human leukocyte antigens (HLAs). B-cell epitopes were predicted using the BepiPred tool. Predicted epitopes underwent docking studies with HLA molecules to evaluate binding properties. In-silico analyses confirmed the antigenicity, promiscuity, and non-glycosylated nature of the selected epitopes.

**Results:**

Several T and B cell epitopes within SpA were identified, showcasing high binding affinities and extensive population coverage. A multi-epitope vaccine construct, linked by synthetic linkers and an adjuvant, was modelled, refined, and validated through various bioinformatics assessments. The vaccine candidate was subsequently docked with Toll-like receptor 4 (TLR-4) to evaluate its potential for immunogenicity.

**Conclusion:**

This study lays the groundwork for developing epitope-based vaccines targeting SpA in MRSA, identifying promising candidates for experimental validation and contributing to innovative immunotherapeutic strategies against MRSA infections.

## 1 Introduction

Methicillin-resistant *Staphylococcus aureus* (MRSA) is a formidable pathogen that represents a significant public health challenge (Turner et al., 2019). Its extensive resistance to conventional antibiotics, combined with its ability to cause a spectrum of severe infections—from localized skin infections to critical conditions such as pneumonia, endocarditis, and sepsis—positions MRSA as a top priority for healthcare systems worldwide (Denissen et al., 2022). Globally, MRSA contributes to a substantial burden of morbidity and mortality, ranking among the top six multidrug-resistant (MDR) bacteria responsible for an estimated 700,000 deaths annually due to antibiotic resistance (GBD 2021 Antimicrobial Resistance Collaborators, 2024). The persistence and adaptability of MRSA necessitate the exploration of novel therapeutic strategies beyond traditional antibiotic treatment (Parsons et al., 2023). One promising avenue in the fight against MRSA is the development of epitope-based vaccines. Epitopes are specific regions of antigens recognized by the immune system, and their precise targeting is crucial for eliciting effective immune responses (Klimka et al., 2021). Such vaccines can specifically target key virulence factors of the bacterium, thereby neutralizing its pathogenic mechanisms. Recent studies have highlighted the importance of immunogenic epitope identification for developing vaccines against resistant pathogens like MRSA, emphasizing the need for innovative approaches to immunotherapy (Khalid and Poh, 2023) *Staphylococcal* Protein A (SpA) emerges as a pivotal virulence factor, exerting profound influence over the host immune response, notably by functioning as a B cell superantigen (Mandelli et al., 2024). SpA has also been implicated in MRSA’s ability to evade both innate and adaptive immune responses, which makes it an attractive target for vaccine development (Howden et al., 2023). The identification of immunogenic epitopes within SpA is crucial for designing vaccines that can effectively target MRSA (Clegg et al., 2021). Superantigens constitute a diverse class of proteins with the remarkable ability to robustly engage and activate the immune system in a nonspecific manner (Bear et al., 2023). Within the arsenal of *Staphylococcus aureus* (*S. aureus*), the production of superantigens serves as a formidable shield, hindering the accurate identification and neutralization of other *S. aureus* antigens by the human immune system. Recent advances in structural biology and immunology have provided new insights into how SpA modulates the immune response, suggesting potential sites for targeted vaccine intervention (Bear et al., 2023). In light of its pivotal role in impeding the generation of effective antibodies and subverting the full functionality of B-cell responses against *S. aureus*, SpA has emerged as a focal point of considerable interest in deciphering the evolutionary dynamics of the human immune response to *S. aureus* (Cruz et al., 2021; Bear et al., 2023). Given its pivotal role in immune evasion, SpA stands as an enticing target for the development of vaccines (Forsgren and Sjöquist, 1966). The targeting of SpA epitopes represents a promising strategy to enhance immune recognition and clearance of MRSA, particularly in the context of multidrug resistance (Pauli et al., 2014). Identifying immunogenic epitopes within SpA can lead to the design of a vaccine that elicits robust and specific immune responses. T-cell epitopes can activate cellular immunity by presenting peptides to T cells through HLA molecules, while B cell epitopes can induce humoral immunity by stimulating antibody production (Jawa et al., 2020). This computational approach accelerates the vaccine development process by streamlining the identification of potential candidates for further experimental validation (Sanchez-Trincado et al., 2017). In this study, we aimed to employ an in-depth immuno-informatics approach to pinpoint potential T&B cell epitopes in SpA protein. By elucidating these epitopes, we aimed not only to contribute to the expanding repertoire of knowledge in the field of vaccine design but also to chart a novel trajectory in the combat against MRSA infections through the application of innovative immunological strategies. The hypothesis driving this work is that a computational identification of SpA-derived epitopes capable of generating both humoral and cellular immune responses can provide valuable candidates for future MRSA vaccine development. We specifically tried to answer the research question: Can potential epitopes in SpA protein capable of generating humoral and cellular immune response be accurately identified against MRSA? The identified epitopes present promising candidates for further experimental validation and eventual vaccine development, which could provide a powerful tool in the ongoing battle against antibiotic-resistant pathogens.

## 2 Methodology

### 2.1 *Staphylococcal* Protein A assessment

The protein sequence of SpA was obtained from the NCBI database https://www.ncbi.nlm.nih.gov/ bearing ID: WP_033567379. To ensure comprehensive coverage and variability analysis, the BLASTP tool was used to extract approximately 250 homologous sequences. Multiple sequence alignment (MSA) was conducted utilizing the COBALT tool available at NCBI https://www.ncbi.nlm.nih.gov/tools/cobalt/re_cobalt.cgi (Papadopoulos and Agarwala, 2007) to identify conserved and variable regions within the protein, which is crucial for epitope prediction. The ProtParam tool https://web.expasy.org/protparam/ was utilized to examine the physicochemical properties of SpA (Wilkins et al., 1999). The physicochemical properties help in understanding the stability and solubility of the protein, which are important for vaccine design.

### 2.2 Epitope screening

#### 2.2.1 T&B cell epitope prediction

Epitopes specific to different Major Histocompatibility Complex (MHC) classes were forecasted using Tepitool http://tools.iedb.org/tepitool/ (Paul et al., 2016) [7]. The HLA Class I alleles (including A & B alleles) were screened using a panel of 27 most common alleles provided in the Tepitool server (**Supplementary Figure 1A**). Peptide lengths were selected as 9mers. To predict HTL epitopes, a panel of 26 most frequent alleles (**Supplementary Figure 1B**) were selected, and the length of peptides selected were 15mers. The peptides with percentile rank ≤ 1 were selected. For the prediction of B cell epitopes, the BepiPred 2.0 tool https://services.healthtech.dtu.dk/services/BepiPred-2.0/ was utilized (Jespersen et al., 2017). All tools were run at default parameters without altering anything.

### 2.3 Epitope selection criteria

Epitopes were selected based on several criteria:

#### 2.3.1 Binding affinity

Epitopes were identified for their robust projected binding affinity to widely occurring HLA alleles. Consequently, only epitopes with higher percentile scores were included for further analysis.

#### 2.3.2 Population coverage

In order to ensure broad applicability across different populations, ensuring the epitopes applicability in diverse demographic groups. For this, we selected only those epitopes which were promiscuous.

#### 2.3.3 Antigenicity

Evaluated using the VaxiJen tool https://www.ddg-pharmfac.net/vaxijen/VaxiJen/VaxiJen.html to predict the potential to be recognized as an antigen (Salod and Mahomed, 2022, pp. 2017–2021).

### 2.4 Molecular docking

Predicted epitopes (CTL & HTL) were subjected to molecular docking studies to assess their binding affinities with HLA molecules. ClusPro tool https://cluspro.org/help.phpb was used for these studies (Vajda et al., 2017). The binding interactions were visualized and the interaction patterns were analyzed using Pymol to ensure strong and specific binding.

### 2.5 Constructing a multi-epitope chain and its interaction analysis

The selected T&B-cell epitopes were concatenated into a single multi-epitope chain. Linker sequences were used to join the epitopes to enhance their presentation and processing by the immune system. Furthermore, the vaccine construct was supplemented with an adjuvant and PADRE sequence to bolster its immunogenicity and facilitate epitope presentation. The physicochemical properties of the finalized vaccine construct was also noted. The I-Tasser server https://zhanggroup.org/I-TASSER/ (Zhou et al., 2022) was employed to initially model the multi-epitope chain, followed by refinement utilizing the Galaxy refine server https://galaxy.seoklab.org/cgi-bin/submit.cgi?type=REFINE (Heo et al., 2013). The refined vaccine’s 3D model underwent validation using the ProSA-web tool https://bio.tools/prosa-web (Wiederstein and Sippl, 2007). In addition, the Ramachandran plot was also investigated using MolProbity https://phenix-online.org/documentation/reference/molprobity_tool.html and the structural robustness of the modelled vaccine was examined using CABS-flex 2.0 https://bio.tools/cabs-flex_2.0 (Kuriata et al., 2018). Molecular docking with the Cluspro server https://cluspro.org/help.php was performed to determine the ability of the multi-epitope construct to trigger innate immune activation via TLR-4.

### 2.6 Disulfide engineering

In order to improve the structural stability of the vaccine structure numerous disulfide bonds were incorporated into the refined vaccine construct utilizing the design v2.0 web server (Xie et al., 2018).

### 2.7 Immune simulation

The vaccine sequence was also investigated foe the ability to induce humoral and adaptive immune responses. We employed in silico immune simulation assay for the same utilizing C-immsim server (Stolfi et al., 2022).

## 3 Results

### 3.1 SpA assessment

**Figure 1:**
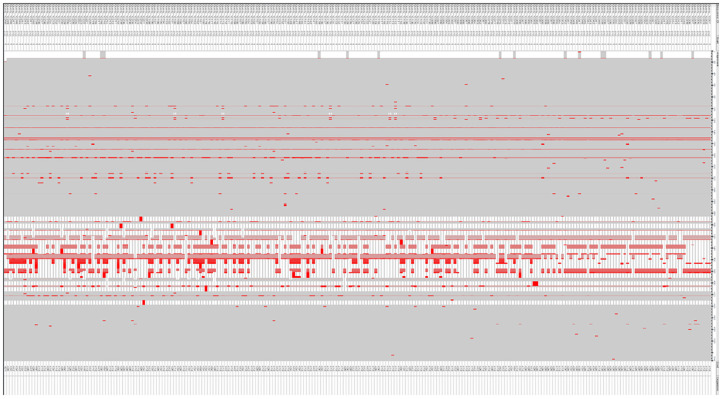
Comparison of Spa protein sequences to detect mutations, conducted through the COBALT tool for multiple sequence alignment.

Multiple sequence alignments revealed some mutations in the Spa protein. These mutations were noted for emitting the epitopes lying within these regions (**Figure 1**). The protein’s properties unveiled its molecular weight at 45528.47, theoretical pI at 5.65, with 56 +vely charged residues and 62-vely charged residues. The projected ½ life spanned 30 hrs (mammalian reticulocytes, *in-vitro*), greater than 20 hours (yeast, *in-vivo*), and greater than 10 hours (*E. coli*, *in-vivo*). The Aliphatic and instability index stood at 69.47, (II) and 50.49 respectively. The GRAVY was measured at -0.991. The identified mutations could potentially impact the immunogenic properties of SpA by altering its epitope structure or antigen presentation. These changes may influence vaccine efficacy, necessitating careful consideration during epitope selection and structural modeling. The molecular weight and pI of the SpA are crucial factors that can influence its immunogenic properties. The molecular weight of a protein impacts its processing and presentation by antigen-presenting cells, as smaller peptides may be more readily processed, while larger proteins may require additional processing steps. The theoretical pI, on the other hand, reflects the protein’s charge at physiological pH, affecting its solubility, stability, and binding interactions with immune receptors and antibodies. Proteins with pI values close to the physiological pH are often more soluble and can interact more effectively with immune components, potentially enhancing their immunogenicity. These properties, therefore, underscore the significance of SpA’s structural and physicochemical characteristics in the design of effective epitope-based vaccines.

### 3.2 Epitope screening

The Tepitool server from IEDB was used to forecast HLA-Class I & II epitopes. Tepitool predicts how peptides bind to HLA molecules, with HLA class II molecules accommodating longer peptides due to their expanded binding groove, in contrast to the confined groove in HLA class I molecules. Despite this difference, the core binding region of class II molecules is typically around 9 amino acids in length. Flanking amino acids enhances the stability of the binding without directly interacting with the HLA binding groove. To maximize the coverage of potential binding cores while minimizing redundancy, we selected 15mer peptides overlapping by 10 amino acids. This approach ensured that all possible 9mer binding cores were represented, with a minimum of one flanking residue on each side for stability. This approach eliminates the selection of overlapping peptides that share identical binding cores (Paul et al., 2016). We followed the IEDB recommended method for predicting and selecting the most ideal peptides, which is the default prediction method in Tepitool. While 15mer peptides were selected for optimal CD4+ T cell recognition, their larger size may lead to overlap with adjacent sequences, potentially reducing specificity and increasing cross-reactivity. Additionally, larger peptides may face processing limitations that could impact overall immunogenicity. However, if we do carry out blast analysis against the human genome, this will reduce the probability of cross reactivity. For HLA Class I epitopes, 9 mers were selected. Tepitool selects the best prediction approach available for a given MHC molecule based on predictor availability and historical performance. Peptides with a percentile rank of less than 0.5 were only selected for further screening. A total of 71 epitopes targeting HLA Class I alleles and 33 promiscuous epitopes targeting HLA Class II alleles were predicted. In addition, BepiPred 2.0 predicted nearly 12 B cell epitopes (**Supplementary Figure 2**). The IEDB database-based tools have earlier been employed by numerous research groups to predict promising epitopes targeting viral (Chauhan et al., 2018; Chauhan and Singh, 2020) bacterial (Ghafouri et al., 2024), parasitic (Chauhan et al., 2023) and other metabolic disorders (Banesh et al., 2024). This underscores the reliability of the predicted epitopes for vaccine applications.

### 3.3 Epitope selection criteria

The selected epitopes underwent a comprehensive filtering process before molecular docking analysis to ensure their suitability for vaccine development. The filters applied and their relevance are discussed below:

#### 3.3.1 High predicted affinities to HLA alleles

The efficacy of an immune response is significantly influenced by how well epitopes bind to HLA molecules. Our results demonstrated that the selected epitopes exhibited strong binding affinities, as indicated by their high percentile scores.

#### 3.3.2 Broad population coverage

Ensuring broad population coverage is imperative for the universal applicability of a vaccine. To achieve this, we selected promiscuous epitopes capable of binding to multiple HLA alleles, thereby covering a larger population. This approach increases the likelihood that a significant proportion of the population will respond to the vaccine, thus enhancing its overall efficacy.

#### 3.3.3 Immunogenicity

Peptide-based vaccines often exhibit lower immunogenicity. To address this, we utilized the VaxiJen server to select epitopes with high immunogenic potential (Doytchinova and Flower, 2007). The chosen epitopes demonstrated the ability to trigger both humoral and cellular immune responses, indicating their effectiveness as vaccine candidates.

#### 3.3.4 Consideration of glycosylation sites

Glycosylation can impact the stability, immunogenicity, and efficacy of epitopes. Using the NetNGlyc tool (Gupta and Brunak, 2002), we excluded epitopes lying within glycosylation regions to ensure optimal performance. This additional filter further refined our selection, enhancing the stability and immune recognition of the epitopes.

#### 3.3.5 Molecular docking

**Figure 2:**
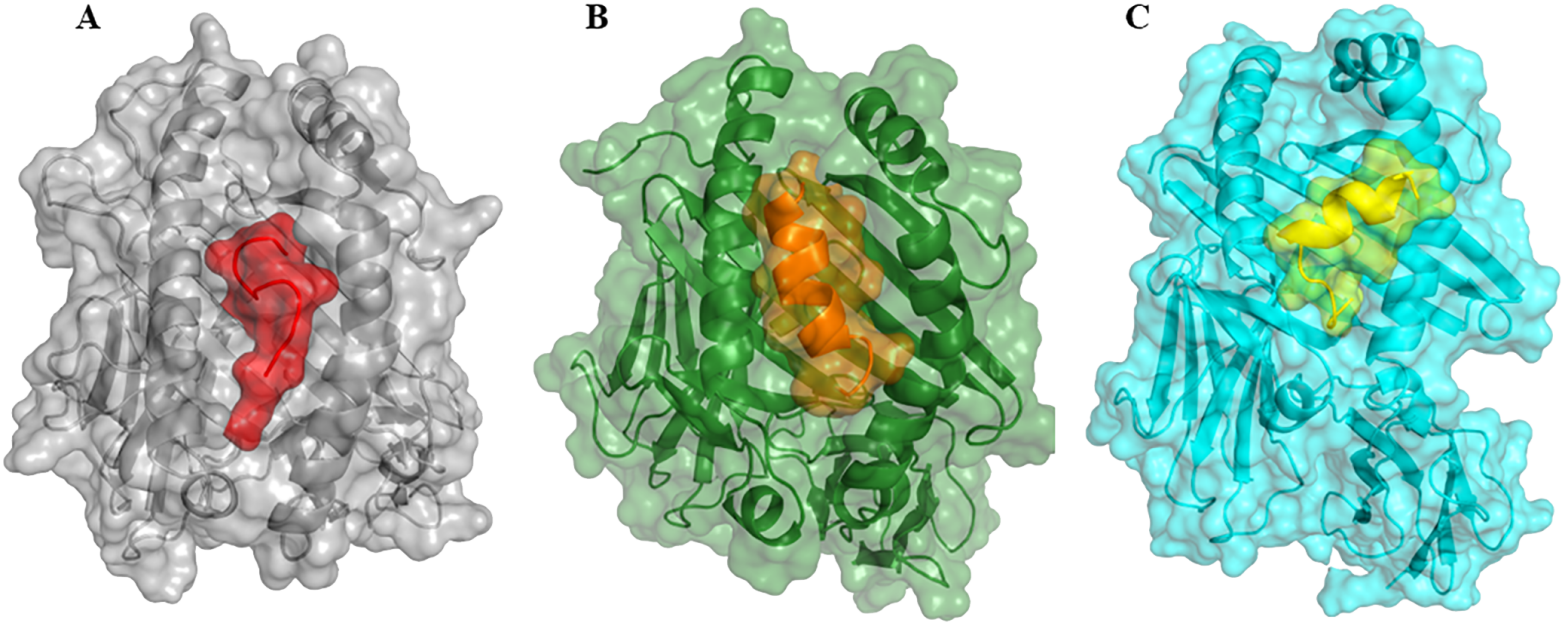
The interaction patterns of epitopes to HLA molecules in cartoon and surface view. **(A)** Interaction of HLA Class I epitopes (represented as red color surface view) to HLA-A*02:01 allele (grey color, surface view). **(B)** Interaction of HLA Class II epitope (orange color, surface view) to HLA-DRB1*01:01 (represented in dark green color, surface view retrieved from PDB with ID: 2g9h). **(C)** Interaction of HLA Class II epitope (yellow color, surface view) to HLA-DRB1*15:01 (cyan color, surface view, retrieved from PDB with ID: 1bx2). Note, that all epitopes interacted with the HLA binding grooves.

The epitopes were further docked with HLA alleles. Visual analysis of the docking complexes revealed that the epitopes fit well into the binding domains of the HLA molecules, forming stable hydrogen bonds with key residues. For HLA Class II-epitope interaction analysis, docking was performed with two HLA-DRB1 alleles i.e. 01 & 15. In contrast, for HLA Class I-epitope interaction analysis, we used the HLA-A*02:01 allele (**Figure 2**). This analysis confirmed that the epitopes could be effectively displayed by HLA molecules on the membrane of antigen-presenting cells.

### 3.4 Epitopes finalization

**Figure 3:**
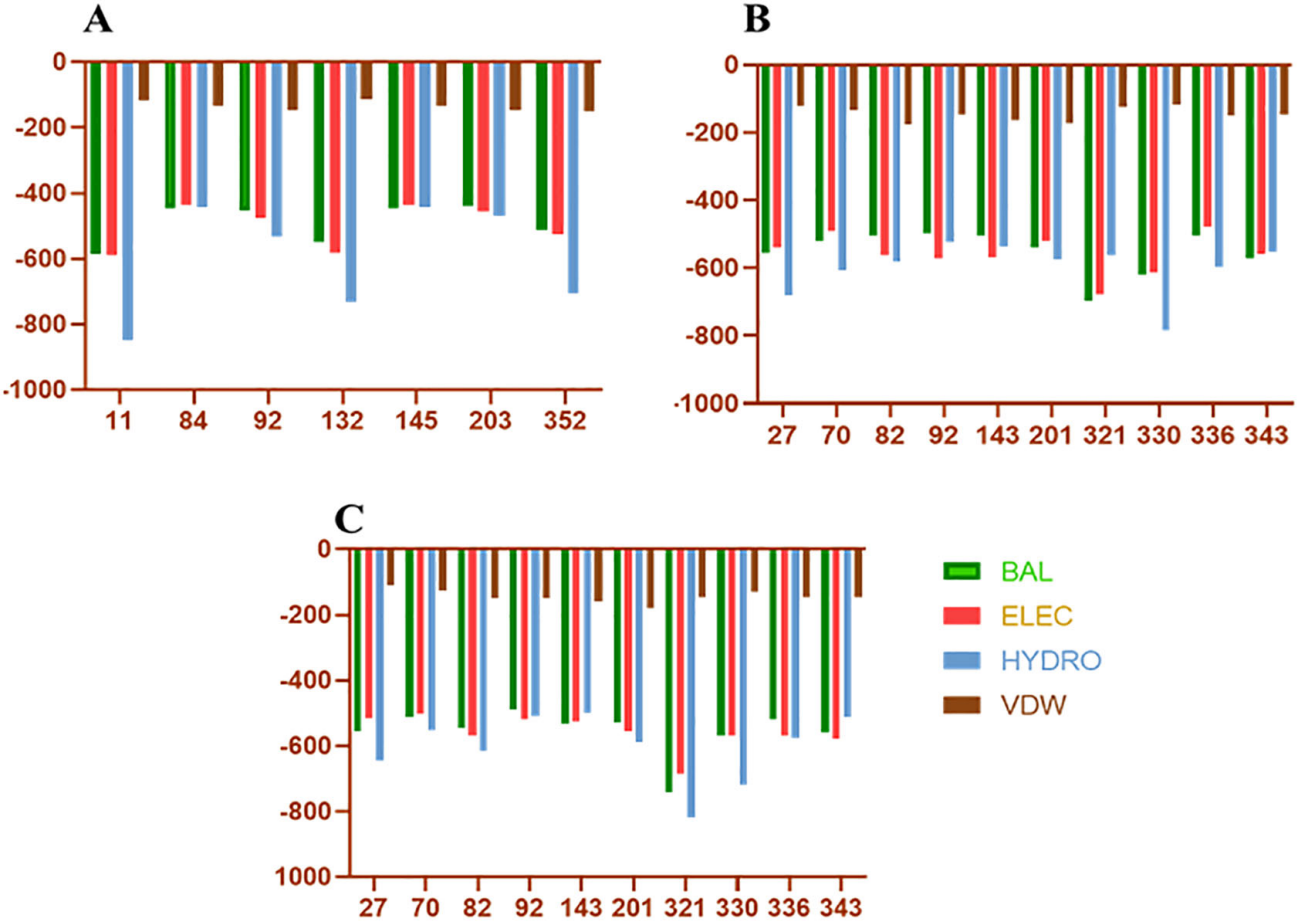
Docking energies of T-cell epitopes included in vaccine with HLA molecules. **(A–C)** Represents the docking energies of the epitopes with different HLA alleles. On the Y-axis are the docking energies and on the X-axis are the position of epitopes. BAL, Balanced energy; ELEC, Electrostatic favored energy; HYDRO, Hydrophobic favored energy; VDW, Vander walls + electrostatic energy.

After applying the above filters, we identified 10 HTL and 7 CTL epitopes. These epitopes demonstrated high binding affinity (**Figure 3**), broad population coverage, and immunogenicity, and were free from glycosylation sites. For the identification of the B cell epitope, we employed the BepiPred 2.0 tool with a threshold of 0.5. We selected epitopes that were above this threshold, greater than 10 amino acids in length, conserved, immunogenic, and not within glycosylation sites. Based on these criteria, 3 B cell epitopes were selected (**Table 1**). The above filters led to the identification of the most promising epitopes within SpA protein. Similar kinds of filters have earlier been employed by several research groups in their immuno-informatics study to screen out epitopes for high success probabilities (Chauhan et al., 2021). The physicochemical properties of the finally selected epitopes are represented in **Supplementary Table 1**.

**Table 1:** Finalized T and B cell epitopes.

Location	Epitope sequence	Interacting HLA alleles	Antigenicity score
HLA Class I epitopes			
11-19	KLGVGIASV	HLA-A*02:01, HLA-A*02:03, HLA-A*02:06	0.9
84-92	KLNDSQAPK	HLA-A*03:01, HLA-A*11:01, HLA-A*30:01,	1.28
92-100	KADAQQNKF	HLA-A*01:01, HLA-B*58:01	1.2
132-140	DPSQSANLL	HLA-B*35:01, HLA-B*51:01, HLA-B*53:01,	0.58
145-153	KLNESQAPK	HLA-A*03:01, HLA-A*11:01, HLA-A*30:01	0.98
203-211	KLNDAQAPK	HLA-A*03:01, HLA-A*11:01, HLA-A*30:01	1.09
352-360	KLADKNMIK	HLA-A*03:01, HLA-A*11:01, HLA-A*30:01	0.7
HLA Class II epitopes			
27-41	SGGVTPAANAAQHDE	HLA-DRB1*04:01, HLA-DRB1*04:05, HLA-DRB1*08:02, HLA-DRB1*09:01, HLA-DRB1*11:01, HLA-DRB1*15:01, HLA-DRB3*02:02, HLA-DRB4*01:01, HLA-DQA1*05:01/DQB1*03:01, HLA-DQA1*05:01/DQB1*02:01, HLA-DQA1*04:01/DQB1*04:02, HLA-DQA1*03:01/DQB1*03:02, HLA-DQA1*01:02/DQB1*06:02,	0.83
70-84	DDPSQSANVLGEAQK	HLA-DQA1*01:02/DQB1*06:02, HLA-DQA1*03:01/DQB1*03:02	0.61
82-96	AQKLNDSQAPKADAQ	HLA-DRB1*01:01, HLA-DRB1*04:01, HLA-DRB1*07:01, HLA-DRB1*09:01, HLA-DRB3*02:02, HLA-DQA1*01:02/DQB1*06:02, HLA-DQA1*05:01/DQB1*03:01,	1.01
92-106	KADAQQNKFNKDQQS	HLA-DPA1*01:03/DPB1*02:01, HLA-DPA1*02:01/DPB1*01:01	1.09
143-157	AKKLNESQAPKADNK	HLA-DRB1*01:01, HLA-DRB1*04:01, HLA-DRB1*07:01, HLA-DRB1*09:01, HLA-DRB1*13:02, HLA-DRB3*02:02 HLA-DRB4*01:01, HLA-DQA1*01:02/DQB1*06:02, HLA-DQA1*04:01/DQB1*04:02, HLA-DQA1*05:01/DQB1*03:01	0.9
201-215	AKKLNDAQAPKADNK	HLA-DRB1*01:01, HLA-DRB1*04:01, HLA-DRB1*08:02, HLA-DRB1*09:01, HLA-DRB1*15:01, HLA-DRB3*02:02 HLA-DRB4*01:01, HLA-DRB5*01:01, HLA-DQA1*01:01/DQB1*05:01, HLA-DQA1*01:02/DQB1*06:02, HLA-DQA1*03:01/DQB1*03:02, HLA-DQA1*04:01/DQB1*04:02, HLA-DQA1*05:01/DQB1*02:01, HLA-DQA1*05:01/DQB1*03:01	0.91
321-335	GNGVHVVKPGDTVND	HLA-DRB4*01:01, HLA-DQA1*04:01/DQB1*04:02, HLA-DQA1*05:01/DQB1*03:01	0.94
330-344	GDTVNDIAKANGTTA	HLA-DRB1*08:02, HLA-DRB1*11:01, HLA-DQA1*01:02/DQB1*06:02	0.55
336-350	IAKANGTTADKIAAD	HLA-DRB1*07:01, HLA-DQA1*01:02/DQB1*06:02, HLA-DQA1*03:01/DQB1*03:02, HLA-DQA1*05:01/DQB1*02:01, HLA-DQA1*05:01/DQB1*03:01	1.3
343-357	TADKIAADNKLADKN	HLA-DRB1*03:01, HLA-DRB1*04:01, HLA-DRB1*04:05, HLA-DRB1*08:02, HLA-DRB1*11:01, HLA-DRB1*13:02, HLA-DRB3*01:01, HLA-DRB3*02:02, HLA-DRB4*01:01HLA-DPA1*02:01/DPB1*01:01, HLA-DPA1*02:01/DPB1*05:01, HLA-DPA1*03:01/DPB1*04:02, HLA-DQA1*01:01/DQB1*05:01, HLA-DQA1*04:01/DQB1*04:02, HLA-DQA1*05:01/DQB1*02:01	0.99
B Cell epitopes			
31-45		TPAANAAQHDEAQQN	1.04
69-92		KDDPSQSANVLGEAQKLNDSQAPK	0.6
260-324		KKLNDAQAPKADNKFNKEQQNAFYEILHLPNLTEEQRNGFIQSLKDDPSVSKEILAEAKKLNDAQAPKEEDNNKPGKEDNNKPGKEDGNKPGKEDNKKPGKEDGNKPGKEDGNKPGKEDGNGV	1.08

Their location, sequence, promiscuousity and antigenicity are depicted.

### 3.6 Multi-epitope vaccine: structural modelling and validation

**Figure 4:**
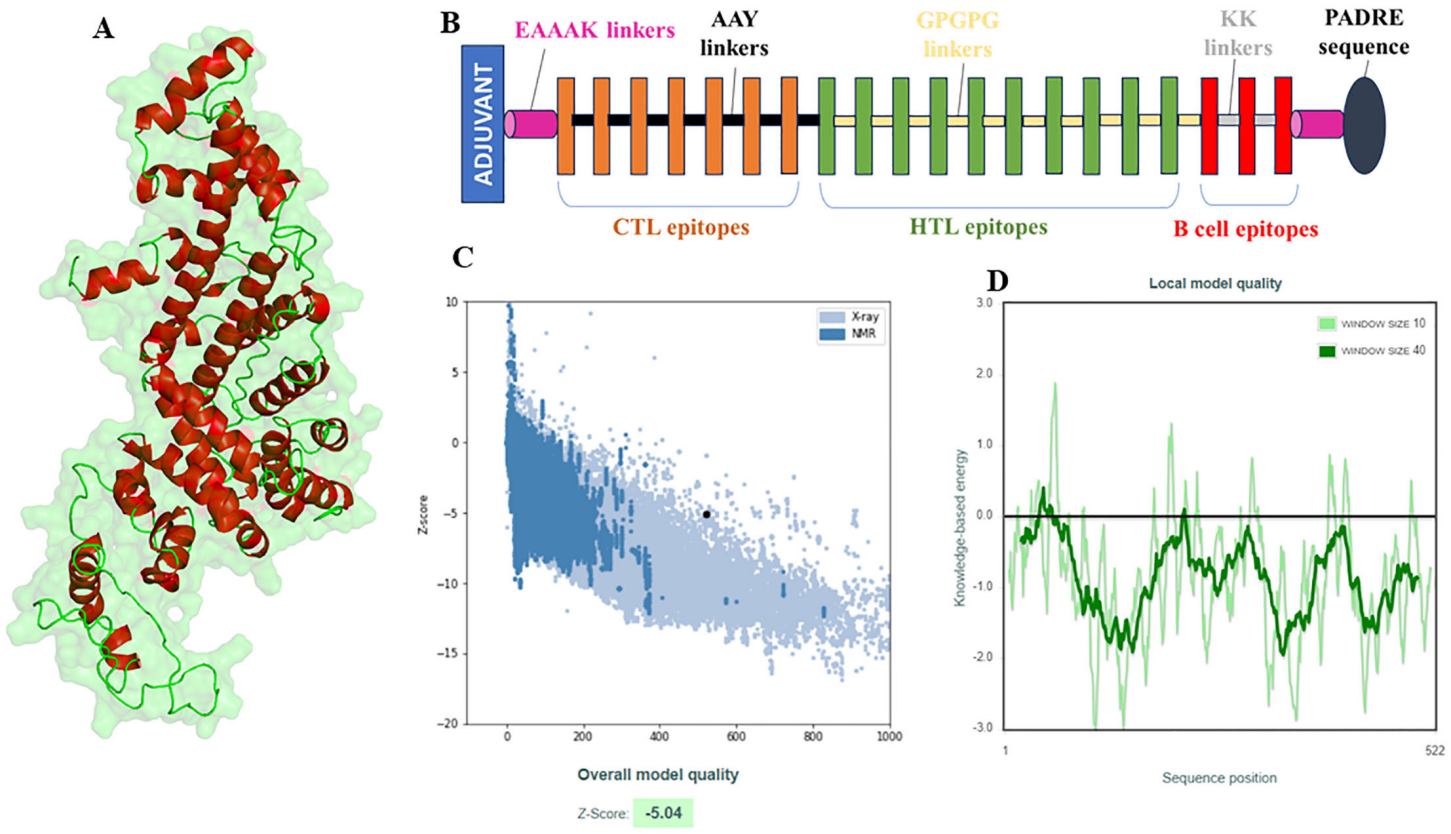
Structural modelling and validation of multi-epitope vaccine construct including initial 3D model, refinement process, and ramachandran plot analysis. **(A)** Visualization of the 3D structure of the vaccine model, **(B)** Demonstration of layout (composition) of the vaccine model., **(C, D)** Structural quality assessment of the constructed and refined 3D model of the vaccine using the ProSA-web tool. It is noteworthy that the Z score falls within the spectrum of proteins with comparable characteristics **(C)**.

**Figure 5:**
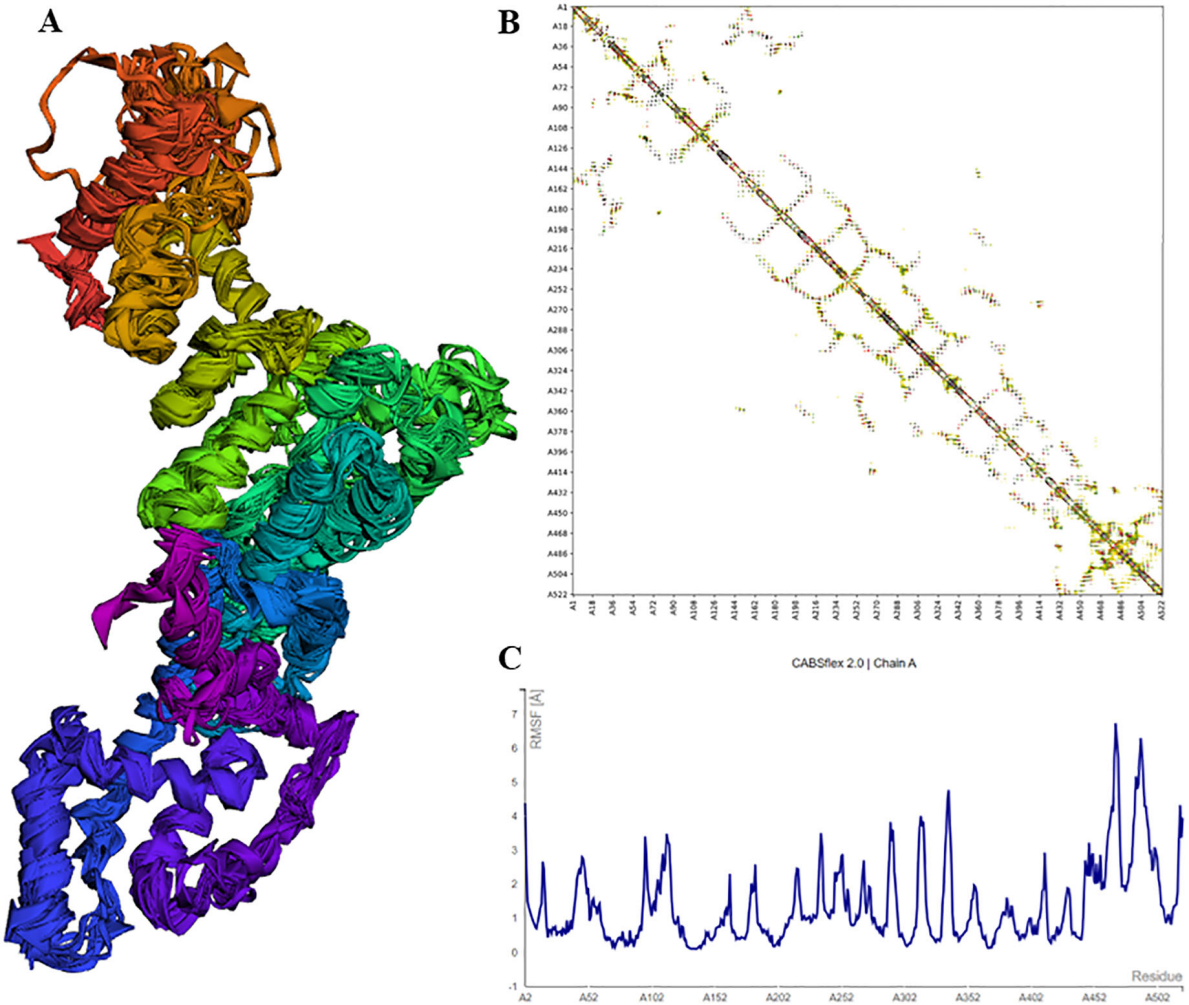
Structural analysis of vaccine model showing flexibility, residue-residue interactions, and RMSF plot indicating fluctuations. **(A)** Cartoon representations of the ten final models show limited overall fluctuations, with notable variations concentrated in the red and brown regions where the adjuvant is linked. **(B)** The contact map offers a comprehensive depiction of residue-residue interactions within the protein, emphasizing interactive regions in the central panel. Particularly noteworthy are the enhanced interactive patterns in the latter portion of the sequence housing the epitopes. **(C)** The fluctuation plot delineates residue fluctuations during the simulation, highlighting increased flexibility in the latter sequence segment.

The vaccine construct was designed by integrating the optimal T & B cell epitopes to induce a strong immune response. The epitopes selected from previous screening stages (10 HTL, 7 CTL, and 3 B cell epitopes) were used as building blocks for these constructs. The vaccine had molecular weight of 53667.56 Daltons, theoretical PI: 5.75, extinction coefficient 15930, estimated half life 30 hours, instability index 32.26, aliphatic index 63.64 and GRAVY -0.796 as predicted by protparam tool. The incorporation of adjuvants and synthetic linkers was aimed at enhancing immunogenicity and ensuring the proper folding of the vaccine constructs. The addition of the 50S ribosomal protein L7/L12 fortified the vaccine construct to enhance the immune response, capitalizing on its robust immunostimulatory capabilities (**Figure 4A**). The initial vaccine constructs were modelled using the I-Tasser server (**Figure 4B**). This tool provided a preliminary 3D structure of the multi-epitope vaccines by predicting the folding and spatial arrangement of the included epitopes. The top 10 templates used by the I-Tasser server for modelling the vaccine construct were 1rquA, 7wkkB, 1rqv, 2nbiA, 1rqv, 8wxbY, 1rquA, 8p0vL, 5h7c and 1dd3A. The model generated had a C-score of -0.97, with estimated TM and RMSD scores as 0.59 ± 0.14 and 9.7 ± 4.6Å respectively. Refinement of the preliminary structure was conducted using the online available Galaxy Refine server. This step was essential to enhance the integrity of the vaccine model by refining both local and global structure, thereby enhancing the overall stability and accuracy of the model. The refinement process included side-chain repacking and overall energy minimization, leading to more reliable and realistic vaccine structures. The refined vaccine models were validated using Ramachandran plot analysis using MolProbilty. The overall amino acids lying in the favored region were 98.5% (**Supplementary Figure 3**). This method assesses the stereochemical characteristics of the protein structures by assessing the φ (phi) and ψ (psi) dihedral angles of the aa residues. It was observed that a predominant number of residues in the refined vaccine constructs were situated within the favored regions of the Ramachandran plot (about 98.5%), indicating a high-quality and reliable structure. The results obtained by the ProSa-web tool also demonstrated the considerable structural integrity of the vaccine construct (**Figures 4C, D**). Finally, employing the CABSflex 2.0 server, the vaccine’s flexible and rigid domains were delineated by molecular motion analysis, within the vaccine construct. Evaluation of the final 3D structures (ten) demonstrated minor changes at the beginning locations, where the adjuvant was connected. Conversely, notable fluctuations were detected close to the terminus. This was the region where a succession of epitopes was affixed using linkers, delineated in dark brown and red sections (**Figure 5A**). Across all 10 models, the contact map illustrated the patterns of residue-residue interactions (**Figure 5B**). According to the RMSF plot, residue fluctuations varied from 0 Å to 7 Å, with more pronounced variability towards the latter part of the sequence, mainly comprising the epitopes, suggesting a high degree of flexibility in these regions (**Figure 5C**).

### 3.6 Molecular docking analysis

**Figure 6:**
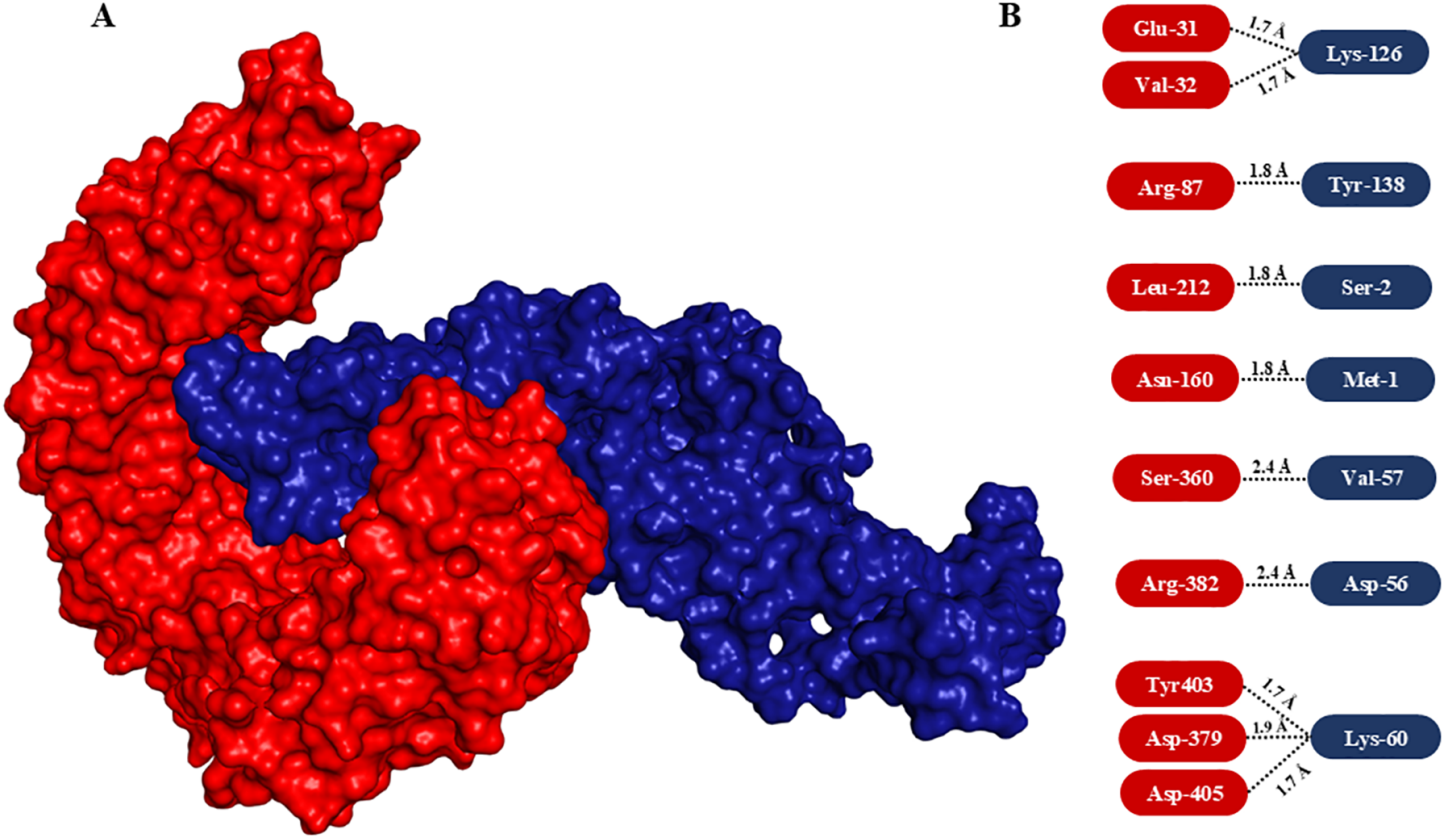
Molecular docking of vaccine construct with TLR-4 and interaction analysis. **(A)** Surface view of the interaction between modelled vaccine (dark blue) with TLR-4 (red). Note the vaccine interacted with the immune receptor nicely by integrating deep into TLR-4. **(B)** Interacted amino acids. The interacting amino acids of TLR-4 and vaccine are highlighted in rectangular rounded corners in red and blue boxes, respectively.

In order to explore the interaction between the vaccine model and TLR-4, molecular docking analysis was performed, aiming to assess its binding affinity and potential immunostimulatory effects. The ClusPro server was employed for the same. It provided a detailed understanding of the binding interactions, depicting binding energies and the interacted amino acids. The vaccine construct exhibited a strong binding affinity to TLR-4, as indicated by the docking score, which revealed stability with significant interactions. Visual analysis of the docking complex demonstrated multiple H-bond interactions between the vaccine and TLR-4. Key residues of TLR-4 involved in binding with the vaccine were identified, ensuring the stability and specificity of the interaction (**Figure 6**). The robust binding conformation of the vaccine construct in the TLR-4 binding pocket hints at effective interaction with the receptor, potentially bolstering immune response through TLR-4-mediated signaling pathways. This strong and stable binding interaction with TLR-4 is crucial for activating innate and adaptive immune responses. The presence of an adjuvant in the vaccine construct may significantly contribute to the enhanced immunogenicity, acting as a potent immunostimulant and enhancing interaction with TLR-4. Overall, the stable and specific interactions observed in the docking studies highlight the suitability of the construct for further experimental validation as an effective vaccine against MRSA. These findings support the potential of the proposed vaccine construct to engage TLR-4 and promote an enhanced immune response through the activation of TLR-4-dependent pathways.

### 3.7 Disulfide engineering

**Figure 7:**
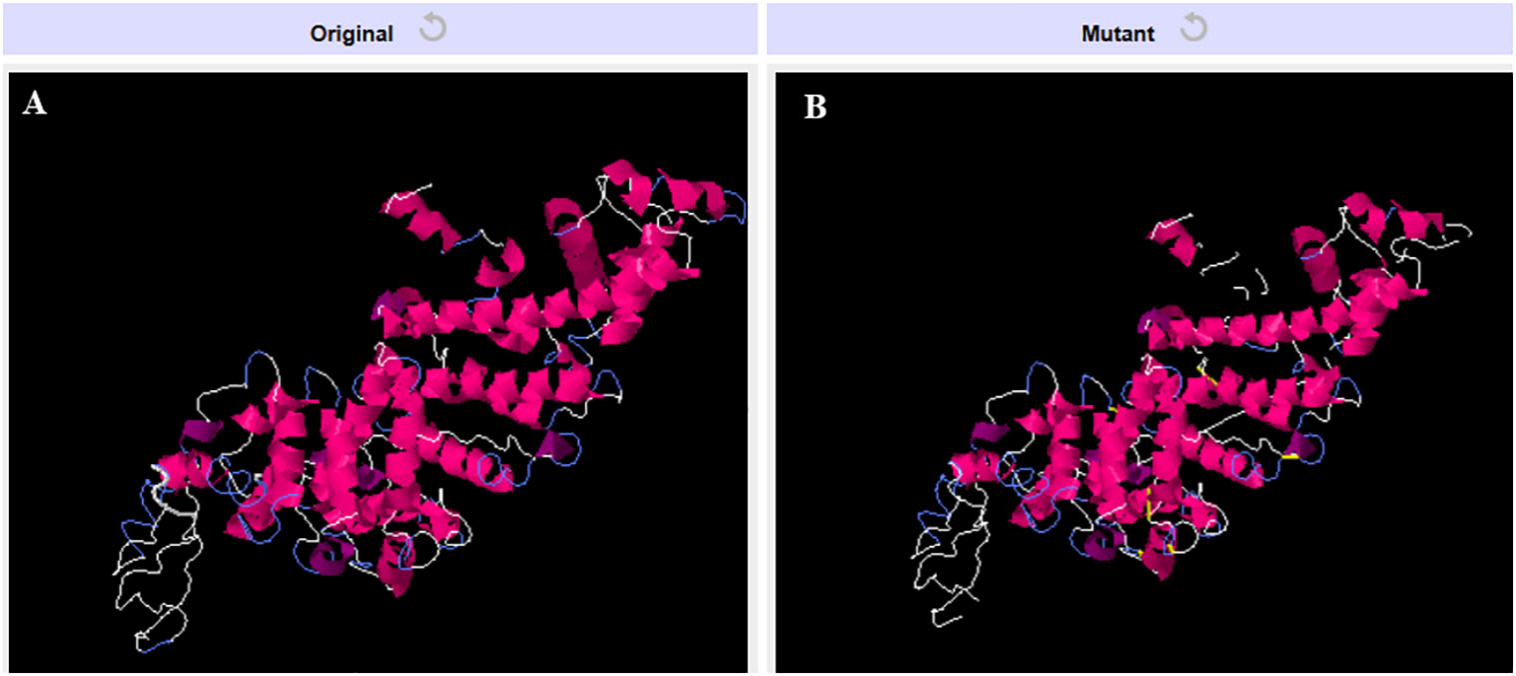
Disulfide engineering of the vaccine construct. **(A)** Original model **(B)** Model generated after disulfide engineering (disulfide bonds highlighted in yellow sticks).

In order to enhance the structural integrity of the vaccine construct, it was subjected to disulfide engineering. Disulfide by Design 2.12 was used for the same. We uploaded the refined model of the vaccine construct to the server and used for residue pair discovery before being used for disulfide engineering. Following that, 18 latent amino acid pairs were shortlisted (**Figure 7**).

### 3.8 Immune simulation assay

**Figure 8:**
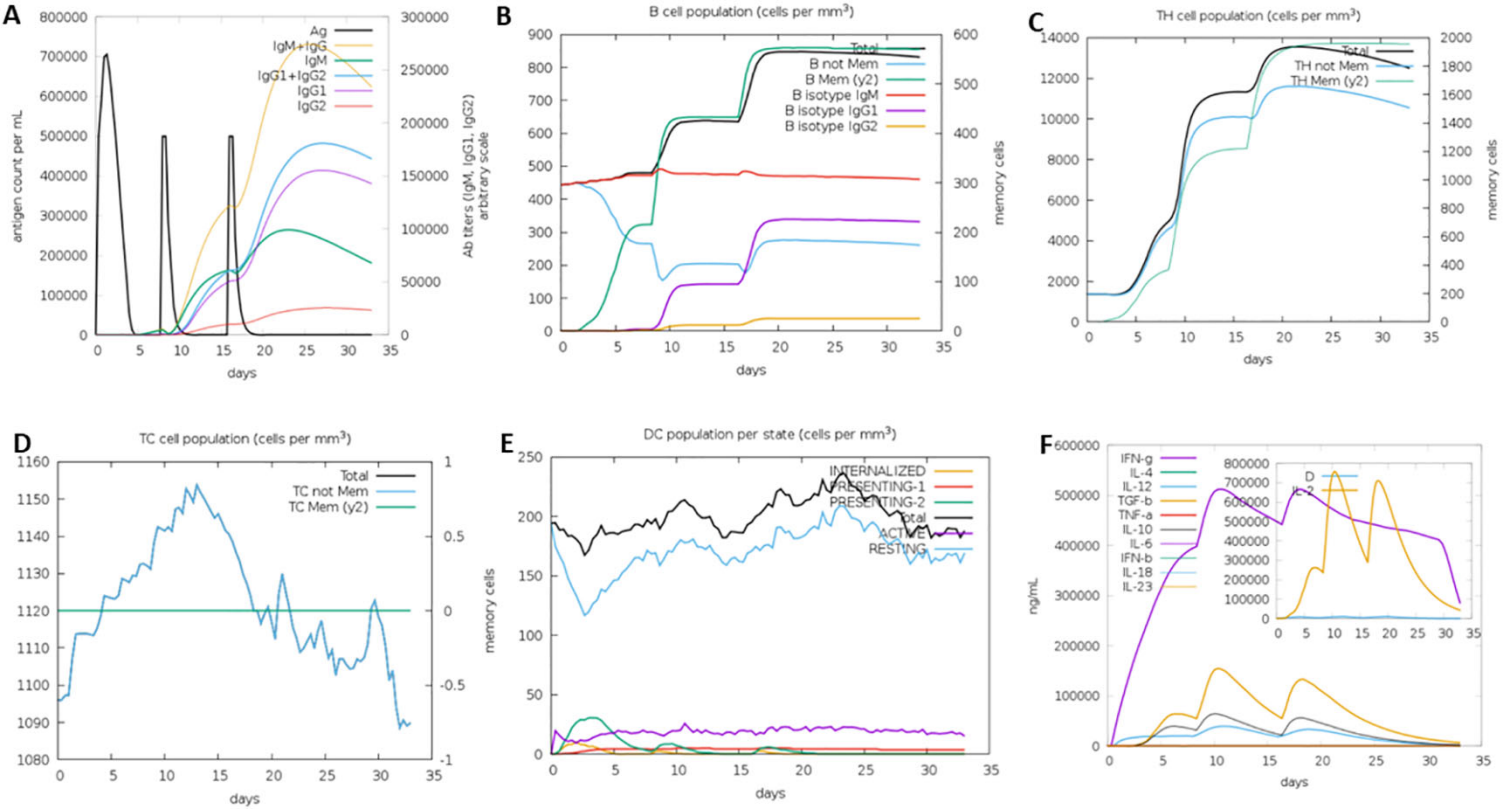
Immune simulation analysis results showing activation of different immune cells: **(A)** Immunoglobulins generation, **(B)** B cell population, **(C)** T helper cells generation, **(D)** Cytotoxic cells generation, **(E)** Dendritic cells generation, and **(F)** Interleukins generation.

The immune simulation was carried out at random seed, simulation volume 10, and number of steps 100, 3 injections at 1-, 24- and 48-weeks interval. The vaccine sequence was observed to elicit varied immune cells revealing its potential immune system activation capability (**Figure 8**).

## 4 Discussion

MRSA possess high resistance to conventional antibiotics and has the ability to cause severe infections ranging from skin infections to life-threatening conditions such as pneumonia and sepsis. The global rise in MRSA infections emphasizes the urgent need for innovative therapeutic strategies, particularly vaccines that prevent infection. While there is currently no licensed vaccine for MRSA, several candidates have been investigated, including protein-based and polysaccharide-conjugate vaccines. However, these approaches have demonstrated limited efficacy in clinical trials, underscoring the necessity for alternative strategies (Miller et al., 2020). The growing incidence of MRSA infections underscores the urgent need for novel therapeutic strategies, particularly those aimed at preventing infections through vaccination (Guo et al., 2020). This study aimed to leverage immuno-informatics to identify potential immunogenic epitopes in SpA a key virulence factor in MRSA, which could serve as a promising target for vaccine development. Tepitool, one of the most advanced tools for predicting epitopes was employed for predicting how peptides bind to HLA molecules, with HLA class II molecules accommodating longer peptides due to their expanded binding groove, in contrast to the confined groove in HLA class I molecules. Despite this difference, the core binding region of class II molecules is typically around 9 amino acids in length. Flanking amino acids enhances the stability of the binding without directly interacting with the HLA binding groove. To maximize the coverage of potential binding cores while minimizing redundancy, we selected 15mer peptides overlapping by 10 amino acids. This approach ensured that all possible 9mer binding cores were represented, with a minimum of one flanking residue on each side for stability. This approach eliminates the selection of overlapping peptides that share identical binding cores (Paul et al., 2016). The same tool and strategy were applied earlier by Moustafa et al. for cell epitope selection (Moustafa et al., 2023). We then filtered the epitopes based on their high predicted affinities to HLA alleles, indicated by high percentile scores. Epitopes with broad population coverage due to their promiscuous nature, immunogenic characteristics, and positions in non-glycosylation sites were prioritized. The present epitope filtration strategy was applied as described earlier (Chauhan et al., 2021). After applying these criteria, the selected epitopes underwent molecular docking, and those demonstrating promising interactions were finally chosen. Finally, we designed the vaccine model concatenated with epitopes which passed stringent filters as applied, linkers, adjuvant (50S ribosomal protein L7/L12) and PADRE sequence. The adjuvant has proven TLR-4 stimulating properties. Studies have shown that incorporating ribosomal proteins as adjuvants can lead to improved vaccine efficacy by promoting antigen presentation. Recently, a similar kind of immuno-informatics study was conducted where the multi-epitope vaccine was developed against *Mycobacterium tuberculosis* and they incorporated 50S ribosomal protein L7/L12 as an immune stimulator in their vaccine sequence (Khan et al., 2023). I-Tasser server produced the 3-D model which we refined and checked its robustness using several validation parameters. Later on, the vaccine model was docked with TLR-4 to examine its binding nature. The presented vaccine has the potential to elicit both humoral and cellular immunity as it is comprised of carefully selected epitopes (cytotoxic, helper T cell and B cell), adjuvant and PADRE sequences. This approach is particularly relevant in the context of the ongoing global effort to combat antibiotic-resistant bacteria through innovative immunological strategies.

Moving forward, *in vitro* and *in vivo* validation studies will be essential to confirm the immunogenicity, safety, and efficacy of the proposed multi-epitope construct. Further experimental testing will allow us to evaluate the immune responses generated by the vaccine, ensuring that it provides adequate protection against MRSA without adverse effects. In addition to validation, future research could benefit from exploring advanced adjuvant formulations and delivery systems to optimize the vaccine’s immunogenicity and stability. Studies investigating this vaccine construct’s effectiveness across diverse population groups are also warranted, given the global prevalence of MRSA and the need for a broadly applicable solution. Such population studies could help tailor the vaccine to address regional HLA variability, enhancing its efficacy and coverage.

## 5 Conclusion

The study successfully identified and screened multiple T & B-cell epitopes within the SpA of MRSA, utilizing advanced immuno-informatics tools. These epitopes were incorporated into multi-epitope vaccine constructs, modelled, and refined to ensure structural stability and immunogenicity. Molecular docking studies demonstrated strong and stable interactions with TLR-4, suggesting the potential for robust immune activation. The study highlights the need for *in vitro* immunogenicity assays to validate epitope-specific responses, animal model studies to assess the safety and efficacy of the multi-epitope vaccine, and adjuvant optimization to enhance immune responses, paving the way for advanced vaccine development against MRSA. The study underscores the potential of epitope-based vaccines targeting SpA as a novel approach to combat MRSA infections.

## Data Availability

Publicly available datasets were analyzed in this study. This data can be found here: NCBI - WP_033567379.

## Supplementary material

Supplementary Figure 1 HLA Class I **(A)** and Class II alleles **(B)** targeted for epitope prediction.

Supplementary Figure 2 Overall results of BepiPred-2.0 server.

Supplementary Figure 3 Ramachandran Plot of the refined vaccine construct. 98.5% of residues were in the favored region.
