# Supplementary material for: Innovative epitopes in Staphylococcal Protein-A an immuno-informatics approach to combat MDR-MRSA infections
Source: Front Cell Infect Microbiol. 2025 Jan 14;14:1503944. doi: 10.3389/fcimb.2024.1503944 (PMC11772303; doi:10.3389/fcimb.2024.1503944)
Supplement: Supplementary Figure 1 — HLA Class I (A) and Class II alleles (B) targeted for epitope prediction. [file SupplementaryFile1.docx]

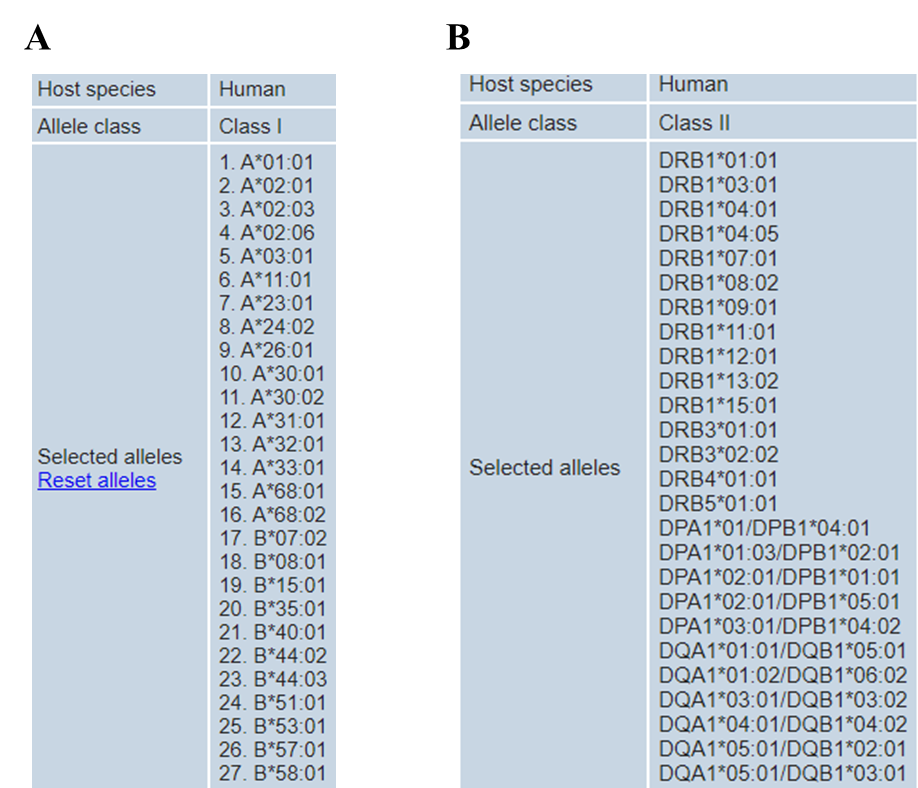


**Figure S 1:** List of HLA Class I (A) and Class II (B) alleles targeted for epitope prediction.


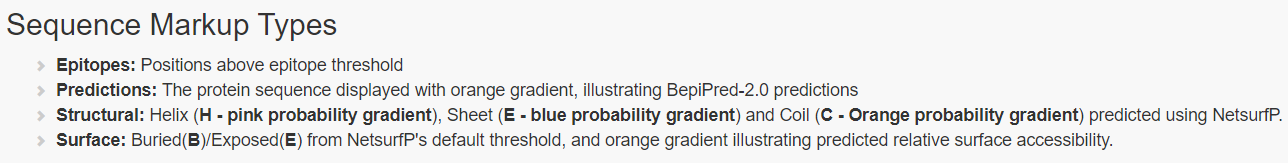


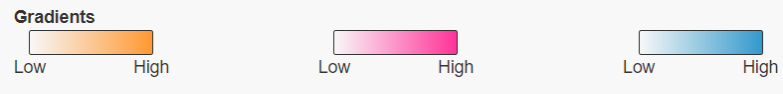

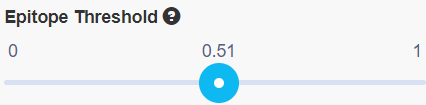


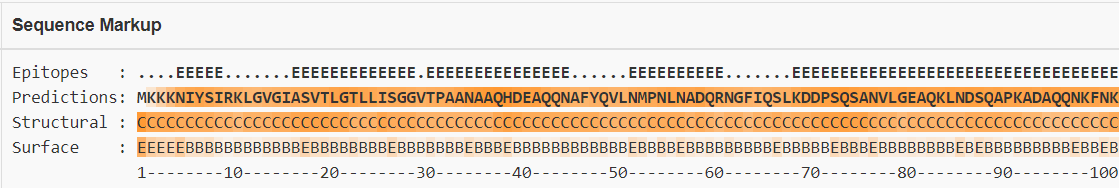

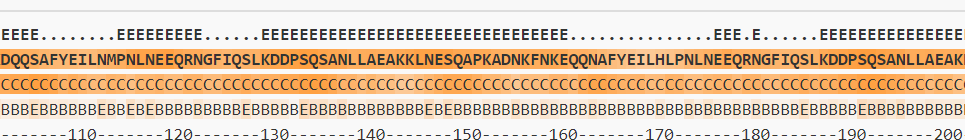


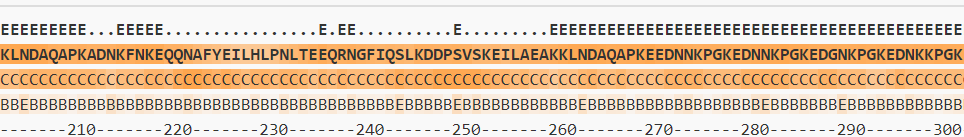


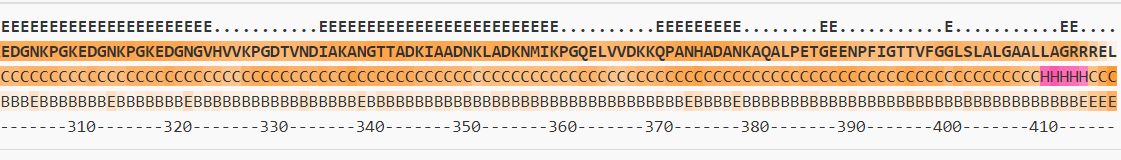


**Figure S 2:** Overall results of BepiPred-2.0 server.

**Table S 1.** Finalised T and B cell epitopes. Their location, sequence, promiscuousity and antigenicity are depicted.

| **Location** | **Epitope sequence** | **Interacting HLA alleles** | **Antigenicity score** |
| --- | --- | --- | --- |
| **HLA Class I epitopes** | | | |
| 11-19 | KLGVGIASV | HLA-A*02:01, HLA-A*02:03, HLA-A*02:06 | 0.9 |
| 84-92 | KLNDSQAPK | HLA-A*03:01, HLA-A*11:01, HLA-A*30:01 | 1.28 |
| 92-100 | KADAQQNKF | HLA-A*01:01, HLA-B*58:01 | 1.2 |
| 132-140 | DPSQSANLL | HLA-B*35:01, HLA-B*51:01, HLA-B*53:01 | 0.58 |
| 145-153 | KLNESQAPK | HLA-A*03:01, HLA-A*11:01, HLA-A*30:01 | 0.98 |
| 203-211 | KLNDAQAPK | HLA-A*03:01, HLA-A*11:01, HLA-A*30:01 | 1.09 |
| 352-360 | KLADKNMIK | HLA-A*03:01, HLA-A*11:01, HLA-A*30:01 | 0.7 |
| **HLA Class II epitopes** | | | |
| 27-41 | SGGVTPAANAAQHDE | HLA-DRB1*04:01, HLA-DRB1*04:05, HLA-DRB1*08:02, HLA-DRB1*09:01, HLA-DRB1*11:01, HLA-DRB1*15:01, HLA-DRB3*02:02, HLA-DRB4*01:01, HLA-DQA1*05:01/DQB1*03:01, HLA-DQA1*05:01/DQB1*02:01, HLA-DQA1*04:01/DQB1*04:02, HLA-DQA1*03:01/DQB1*03:02, HLA-DQA1*01:02/DQB1*06:02 | 0.83 |
| 70-84 | DDPSQSANVLGEAQK | HLA-DQA1*01:02/DQB1*06:02, HLA-DQA1*03:01/DQB1*03:02 | 0.61 |
| 82-96 | AQKLNDSQAPKADAQ | HLA-DRB1*01:01, HLA-DRB1*04:01, HLA-DRB1*07:01, HLA-DRB1*09:01, HLA-DRB3*02:02, HLA-DQA1*01:02/DQB1*06:02, HLA-DQA1*05:01/DQB1*03:01 | 1.01 |
| 92-106 | KADAQQNKFNKDQQS | HLA-DPA1*01:03/DPB1*02:01, HLA-DPA1*02:01/DPB1*01:01 | 1.09 |
| 143-157 | AKKLNESQAPKADNK | HLA-DRB1*01:01, HLA-DRB1*04:01, HLA-DRB1*07:01, HLA-DRB1*09:01, HLA-DRB1*13:02, HLA-DRB3*02:02  HLA-DRB4*01:01, HLA-DQA1*01:02/DQB1*06:02, HLA-DQA1*04:01/DQB1*04:02, HLA-DQA1*05:01/DQB1*03:01 | 0.9 |
| 201-215 | AKKLNDAQAPKADNK | HLA-DRB1*01:01, HLA-DRB1*04:01, HLA-DRB1*08:02, HLA-DRB1*09:01, HLA-DRB1*15:01, HLA-DRB3*02:02  HLA-DRB4*01:01, HLA-DRB5*01:01, HLA-DQA1*01:01/DQB1*05:01, HLA-DQA1*01:02/DQB1*06:02, HLA-DQA1*03:01/DQB1*03:02, HLA-DQA1*04:01/DQB1*04:02, HLA-DQA1*05:01/DQB1*02:01, HLA-DQA1*05:01/DQB1*03:01 | 0.91 |
| 321-335 | GNGVHVVKPGDTVND | HLA-DRB4*01:01, HLA-DQA1*04:01/DQB1*04:02, HLA-DQA1*05:01/DQB1*03:01 | 0.94 |
| 330-344 | GDTVNDIAKANGTTA | HLA-DRB1*08:02, HLA-DRB1*11:01, HLA-DQA1*01:02/DQB1*06:02 | 0.55 |
| 336-350 | IAKANGTTADKIAAD | HLA-DRB1*07:01, HLA-DQA1*01:02/DQB1*06:02, HLA-DQA1*03:01/DQB1*03:02, HLA-DQA1*05:01/DQB1*02:01, HLA-DQA1*05:01/DQB1*03:01 | 1.3 |
| 343-357 | TADKIAADNKLADKN | HLA-DRB1*03:01, HLA-DRB1*04:01, HLA-DRB1*04:05, HLA-DRB1*08:02, HLA-DRB1*11:01, HLA-DRB1*13:02, HLA-DRB3*01:01, HLA-DRB3*02:02, HLA-DRB4*01:01HLA-DPA1*02:01/DPB1*01:01, HLA-DPA1*02:01/DPB1*05:01, HLA-DPA1*03:01/DPB1*04:02, HLA-DQA1*01:01/DQB1*05:01, HLA-DQA1*04:01/DQB1*04:02, HLA-DQA1*05:01/DQB1*02:01 | 0.99 |
|  |  | **B Cell epitopes** |  |
| 31-45 | TPAANAAQHDEAQQN | | 1.04 |
| 69-92 | KDDPSQSANVLGEAQKLNDSQAPK | | 0.6 |
| 260-324 | KKLNDAQAPKADNKFNKEQQNAFYEILHLPNLTEEQRNGFIQSLKDDPSVSKEILAEAKKLNDAQAPKEEDNNKPGKEDNNKPGKEDGNKPGKEDNKKPGKEDGNKPGKEDGNKPGKEDGNGV | | 1.08 |

| **Epitope Location** | **Epitope sequence** | **Molecular weight (Daltons)** | **Theoretical pI** | **Extinction coefficients** | **Estimated half-life (mammalian reticulocytes, in vitro)** | **Instability index** | **Aliphatic index** | **Grand average of hydropathicity (GRAVY)** |
| --- | --- | --- | --- | --- | --- | --- | --- | --- |
| **HLA Class I epitopes** | | | | | | | |  |
| 11-19 | KLGVGIASV | 843.03 | 8.75 | - | 1.3 hrs | -19.41 | 162.22 | 1.44 |
| 84-92 | KLNDSQAPK | 1000.12 | 8.59 | - | 1.3 hrs | 63.66 | 54.44 | -1.67 |
| 92-100 | KADAQQNKF | 1049.15 | 8.59 | - | 1.3 hrs | 47.17 | 22.22 | -1.71 |
| 132-140 | DPSQSANLL | 944.01 | 3.80 | - | 1.1 hrs | 100.51 | 97.78 | -0.47 |
| 145-153 | KLNESQAPK | 1014.15 | 8.59 | - | 1.3 hrs | 63.66 | 54.44 | -1.67 |
| 203-211 | KLNDAQAPK | 984.12 | 8.59 | - | 1.3 hrs | 20.86 | 65.56 | -1.38 |
| 352-360 | KLADKNMIK | 1060.32 | 9.70 |  | 1.3 hrs | -28.84 | 97.78 | -0.74 |
| **HLA Class II epitopes** | | | | | | | |  |
| 27-41 | SGGVTPAANAAQHDE | 1424.45 | 4.35 | - | 1.9 hrs | 24.74 | 46.00 | -0.64 |
| 70-84 | DDPSQSANVLGEAQK | 1558.62 | 4.03 | - | 1.1 hrs | 59.28 | 58.67 | -1.12 |
| 82-96 | AQKLNDSQAPKADAQ | 1584.71 | 6.0 | - | 4.4 hrs | 36.53 | 52.67 | -1.34 |
| 92-106 | KADAQQNKFNKDQQS | 1749.86 | 8.50 | - | 1.3 hrs | 90.22 | 13.33 | -2.27 |
| 143-157 | AKKLNESQAPKADNK | 1641.84 | 9.53 | - | 4.4 hrs | 52.32 | 46.0 | -1.7 |
| 201-215 | AKKLNDAQAPKADNK | 1611.82 | 9.53 | - | 4.4 hrs | 26.64 | 52.67 | -1.58 |
| 321-335 | GNGVHVVKPGDTVND | -1507.62 | 5.21 | - | 30 hrs | -23-31 | 77.33 | -0.52 |
| 330-344 | GDTVNDIAKANGTTA | 1447.52 | 4.21 | - | 30 hrs | -16.37 | 65.33 | -0.44 |
| 336-350 | IAKANGTTADKIAAD | 1459.62 | 5.96 | - | 20 hrs | -28.99 | 85.33 | -0.14 |
| 343-357 | TADKIAADNKLADKN |  |  |  |  |  |  |  |
| **B cell epitope** | | | | | | | |  |
| 31-45 | TPAANAAQHDEAQQN | 1587.75 | 5.71 | - | 7.2 hrs | -14.50 | 78.67 | -0.96 |
| 69-92 | KDDPSQSANVLGEAQKLNDSQAPK | 2540.73 | 4.68 | - | 1.3 hrs | 61.34 | 57.08 | -1.33 |
| 260-324 | KKLNDAQAPKADNKFNKEQQNAFYEILHLPNLTEEQRNGFIQSLKDDPSVSKEILAEAKKLNDAQAPKEEDNNKPGKEDNNKPGKEDGNKPGKEDNKKPGKEDGNKPGKEDGNKPGKEDGNGV | 13563.80 | 5.59 | - | 1.3 hrs | 51.20 | 42.93 | -1.78 |

**Table S 2.** Physicochemical properties of the finalised epitopes included in the vaccine construct.


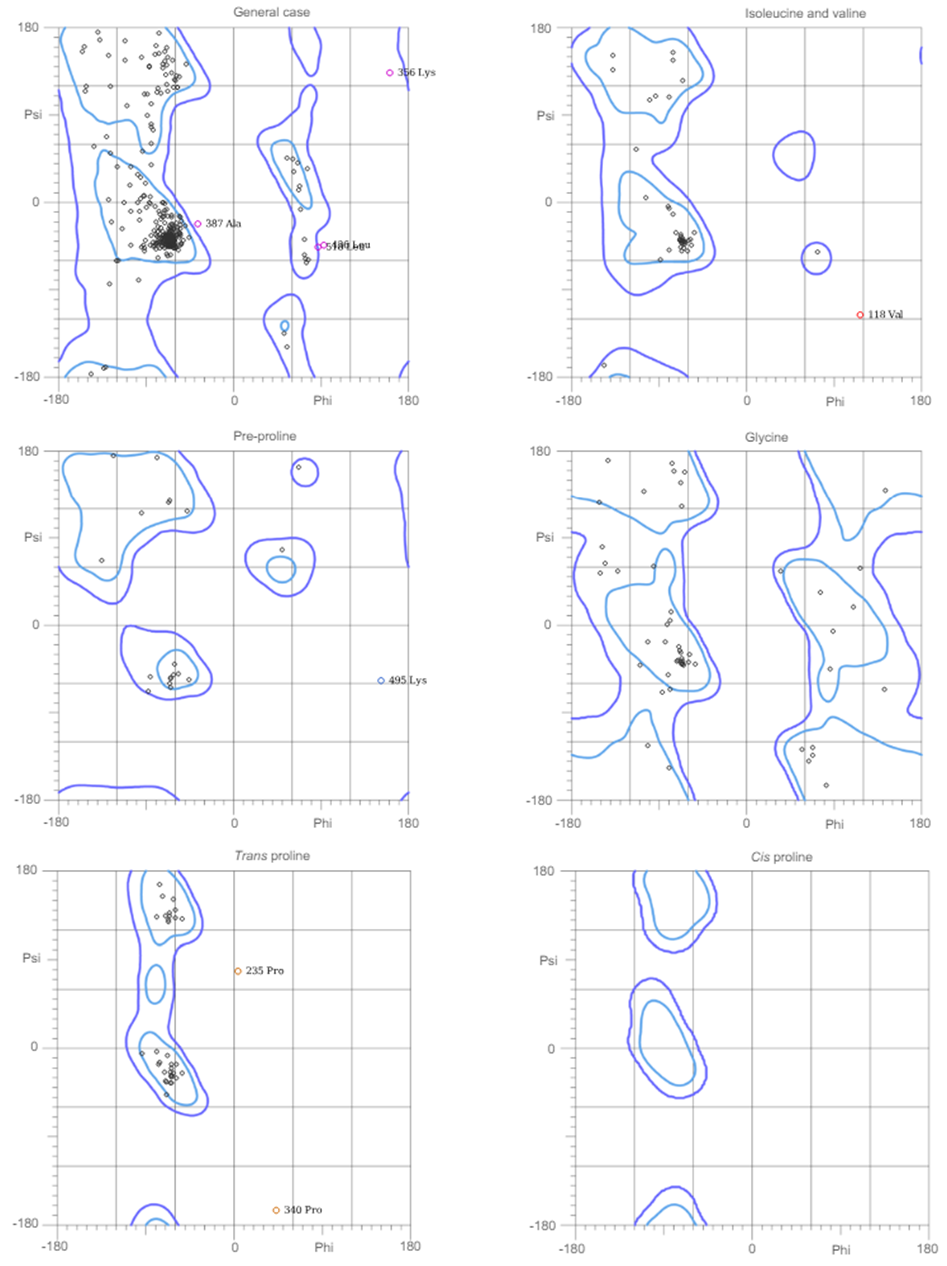


**Figure S 3:** Ramachandran Plot of the refined vaccine construct. 98.5% residues were in favoured region.
